# Study Design of the Microcirculatory Shock Occurrence in Acutely Ill Patients (microSOAP): an International Multicenter Observational Study of Sublingual Microcirculatory Alterations in Intensive Care Patients

**DOI:** 10.1155/2012/121752

**Published:** 2012-05-14

**Authors:** Namkje A. R. Vellinga, E. Christiaan Boerma, Matty Koopmans, Abele Donati, Arnaldo Dubin, Nathan I. Shapiro, Rupert M. Pearse, Jan Bakker, Can Ince

**Affiliations:** ^1^Erasmus MC University Medical Center, Department of Intensive Care Adults, P.O. Box 2040–Room H625, 3000 CA Rotterdam, The Netherlands; ^2^Medical Center Leeuwarden, Department of Intensive Care, P.O. Box 888, 8901 BR Leeuwarden, The Netherlands; ^3^Università Politecnica delle Marche, Department of Biomedical Science and Public Health, 60126 Ancona, Italy; ^4^Sanatorio Otamendi y Miroli, Servicio de Terapia Intensiva, Azcuénaga 870, C1115AAB, Buenos Aires, Argentina; ^5^Beth Isreal Deaconess Medical Center, Department of Emergency Medicine and Center for Vascular Biology Research, 1 Deaconess Road, CC2-W, Boston, MA 02115, USA; ^6^Barts and The London School of Medicine and Dentistry, London, EC1M 6BQ, London, UK

## Abstract

*Objective*. Sublingual microcirculatory alterations are associated with an adverse prognosis in several critical illness subgroups. Up to now, single-center studies have reported on sublingual microcirculatory alterations in ICU patient subgroups, but an extensive evaluation of the prevalence of these alterations is lacking. We present the study design of an international multicenter observational study to investigate the prevalence of microcirculatory alterations in critically ill: the Microcirculatory Shock Occurrence in Acutely ill Patients (microSOAP). *Methods*. 36 ICU's worldwide have participated in this study aiming for inclusion of over 500 evaluable patients. To enable communication and data collection, a website, an Open Clinica 3.0 database, and image uploading software have been designed. A one-session assessment of the sublingual microcirculation using Sidestream Dark Field imaging and data collection on patient characteristics has been performed in every ICU patient >18 years, regardless of underlying disease. Statistical analysis will provide insight in the prevalence and severity of sublingual alterations, its relation to systemic hemodynamic variables, disease, therapy, and outcome. *Conclusion*. This study will be the largest microcirculation study ever performed. It is expected that this study will also establish a basis for future studies related to the microcirculation in critically ill.

## 1. Introduction

The microcirculation plays a pivotal role in oxygen delivery to the tissue [1]. It is believed to be a key player in several disease states, such as sepsis and shock. The development of Orthogonal Polarizing Spectral (OPS) imaging and more recently Sidestream Dark Field (SDF) imaging has enabled bedside imaging of the—predominantly sublingual—microcirculation [2, 3]. Main advantage of SDF/OPS imaging is the ability to visualize true capillary hemodynamics in a noninvasive way at the bedside, thereby providing functional information related to the microcirculation where oxygen delivery to the parenchymal cells takes place. With SDF/OPS imaging, the presence of microcirculatory alterations in different critical care patient subgroups, such as sepsis, heart failure, and major surgery, has been widely explored during the past decade [4–9]. These microcirculatory alterations appear to be associated with an adverse prognosis; they are more severe in nonsurvivors in comparison to survivors in sepsis and heart failure, and are associated with the development of complications in abdominal surgery [4–12]. The aforementioned studies have all shown that microcirculatory alterations are apparent in the presence of more or less normal systemic haemodynamic parameters, thereby stressing the potential importance of the microcirculation as an additional target for resuscitation. Several interventions, ranging from vasoactive drugs and fluid therapy to circulatory assist devices, have been shown to have varying effects on their capacity to influence microcirculatory failure [7, 13–22]. 

Although a randomized controlled clinical trial (RCT) is considered as the highest level of evidence in medical research, the Empirics already realized the importance of observation for gaining a better understanding of diseases [23, 24]. Recent literature acknowledges the advantages of a solid observational study as a powerful tool to include large patient numbers with a variety of backgrounds, making the results easier to extrapolate to daily practice as opposed to RCTs with limited inclusion numbers due to stringent inclusion and exclusion criteria. This has especially been advocated for intensive care patients where several large RCTs fail to demonstrate beneficial effects of interventions. The heterogeneous nature of patients and applied therapy as well as uncertain underlying pathophysiology has been associated with this failure, emphasizing the need for more observational studies in intensive care patients to gain a better understanding of both patient characteristics and effects of interventions [25–29].

In microcirculatory research, the presence and significance of microcirculatory failure has been repeatedly demonstrated in single center studies with a limited number of patients in a variety of different ICU populations. However, a solid estimation of the prevalence of microcirculatory alterations in intensive care patients is not available as yet. Therefore, our aim was to conduct a multicenter observational study to map the prevalence of microcirculatory alterations in intensive care patients, irrespective of their underlying disease, to provide a solid basis for further (interventional) studies. The unique nature of this observational trial will be that it will not only observe the behavior of conventional clinical and hemodynamic variables but will also relate these to the behavior of a completely new unexplored physiological compartment in a multicentral international setting. In this paper we describe the trial design and methods we propose of evaluating the data.

## 2. Methods

Several large multicenter prevalence studies in critical care settings have been conducted, such as the Sepsis Occurrence in Acutely ill Patients (SOAP) study, the European Prevalence of Infection in Intensive Care (EPIC) study and the Columbian internet based Observatorio Nacional de Sepsis Pediátrica (ONASEP) [30–32]. We aimed for a similar study design.

### 2.1. Inclusion of Participating Centers

**Figure 1:**
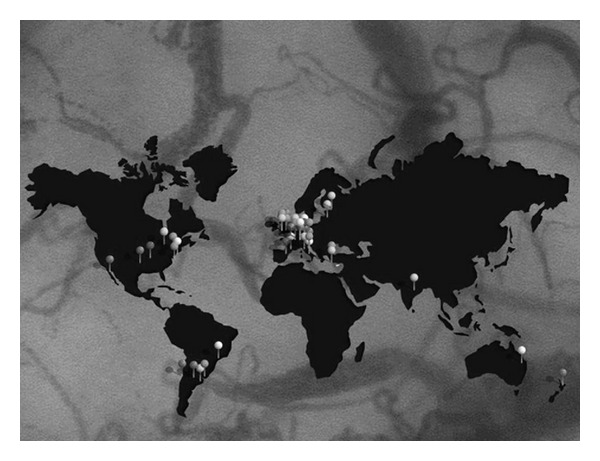
Overview of the ICU's that were invited to participate in the microSOAP.

Out of 47 intensive care units (ICU's) that were invited to participate in this study (see Figure 1 for an overview), 36 ICU's decided to participate.

**The list of participating centers is as follows:**

1. ICU, Medical Center Leeuwarden, Leeuwarden, The Netherlands
2. ICU, Antonius Ziekenhuis, Nieuwegein, The Netherlands
3. ICU, Onze Lieve Vrouwe Gasthuis, Amsterdam, The Netherlands
4. ICU, Erasmus Medical Center, Rotterdam, The Netherlands
5. ICU, Gelre Ziekenhuizen, Apeldoorn, The Netherlands
6. Departamento de Medicina Intensiva, Hospital Clinico de la Pontificia, Universidad Católica de Chile. Santiago, Chili
7. Departamento de Anestesiologia, Dor e terapia Intensiva, Hospital Sao Paulo, Universidade Federal de São Paulo, Sao Paulo, Brasil
8. Servicio de Terapia Intensiva, Sanatorio Otamendi y Miroli, Buenos Aires, Argentina
9. ICU, Hospital San Martín, La Plata, Argentina
10. ICU, Hospital Español “Juan J Crotoggini,” Montevideo, Uruguay
11. ICU, Cooper University Hospital, Camden, USA
12. ICU, Beth Israel Deaconess Medical Center/Harvard Medical School, Boston, USA
13. Critical Care Medicine, University of Pittsburgh, Pittsburgh, PA, USA
14. Critical Care Medicine, St. John's Mercy Medical Center, St Louis, Missouri, USA
15. ICU, University of California, San Diego, USA
16. Universitätsklinikum Jena, Friedrich-Schiller-University, Department of Internal Medicine I, Jena, Germany
17. Department of Surgical Intensive Care, University Hospital Aachen, Aachen, Germany
18. ICU, Royal London Hospital, London, UK
19. ICU, Royal Free Hospital, London, UK
20. ICU, The Royal Marsden Hospital, London, UK
21. ICU, Derriford Hospital and Nuffield Health Plymouth Hospital, Plymouth, UK
22. ICU, New Cross Hospital, Wolverhampton, UK
23. ICU, RDE Hospital, Exeter, UK
24. Critical Care Department, Joan XXIII University Hospital, Tarragona, Spain
25. Department of Intensive Care Medicine, Waikato Hospital, Hamilton, New Zealand
26. ICU, Kaunas University Hospital, Kaunas, Lithuania
27. Clinica di Anestesia e Rianimazione, Azienda Ospedaliera-Universitaria Ospedali Riuniti, Ancona, Italy
28. Dipartimento di Anestesia, Rianimazione e Terapia Intensiva, Azienda ULSS 9 Veneto, Treviso, Italy
29. ICU, Santa Maria degli Angeli Hospital, Pordenone, Italy
30. ICU, Royal Brisbane and Women's Hospital, Brisbane, Australia
31. Departement d'Anesthesie-Reanimation, Hopital de Bicetre, Le Kremlin- Bicêtre, Paris, France
32. Department of Anesthesiology, Critical Care et Samu, Hôpital Lariboisière, Paris, France
33. ICU, University Hospital Basel, Basel, Switzerland
34. Faculty of Tropical Medicine, Mahidol University, Bangkok, Thailand
35. ICU, Hacettepe University, Ankara, Turkey
36. ICU, Kosuyolu University, Istanbul, Turkey.

ICU's were selected based on SDF/OPS availability, established skills in OPS/SDF imaging as demonstrated in a separate teaching course, and/or publications in this field. For image quality check, centers were asked to provide a representative SDF/OPS video of a septic patient and a healthy volunteer, enabling feedback on image quality. During an investigators meeting in March 2011, the study was scheduled for the second week of September 2011. For logistic reasons, it was decided that centers could choose two or more consecutive days for performing measurements. To prevent overlap of patient inclusion, ICU's were divided into (virtual) units and measured as one unit per day. A medical steering committee was formed to oversee the study, including representatives from the different continents as well as the major centers in the participating countries. The medical steering consisted of, E. C. Boerma, MD, PhD; N. A. R. Vellinga, MD; M. Koopmans; A. Donati, MD; A. Dubin, MD, PhD; R. M. Pearse, MD, PhD; N. I. Shapiro, MD, MPH; J. Bakker, MD, PhD; C. Ince, PhD. The study is coordinated from Medical Center Leeuwarden by the principal investigator (E. C. Boerma, MD, PhD), a dedicated physician in charge of running the communication (N. A. R. Velinga, MD) and a research nurse (M. Koopmans). The study center ensures communication with and between study centers, coordinates study logistics, and manages data analysis.

### 2.2. Patient Selection

Every ICU patient ≥18 years, regardless of the underlying disease, was eligible for inclusion. Informed consent was obtained in accordance with local ethics approval. Participation in another study was no exclusion criterion, except when contradictory to local regulations. Patients <18 years or without informed consent were excluded, as well as patients with mucosal bleeding/injury or recent maxillofacial surgery that interfered with SDF/OPS imaging.

### 2.3. Ethics Approval

A study protocol was provided to participating centers. Every participating center obtained ethics approval according to local legislation. A copy of the ethics approval was sent to the study coordinator before start of the study. Written informed consent was obtained of all included subjects, unless the local ethics committee specifically allowed a waiver in this respect.

The study was registered at http://www.clinicaltrials.gov/ (NCT01179243). No (industry) sponsorship has been received for this investigator-initiated study, with the exception of a local hospital fund.

### 2.4. SDF/OPS Imaging

Sublingual OPS and SDF imagings are used for microcirculatory imaging at the bedside with the potential of quantification both at the bedside and offline [33–35]. In short, the OPS and SDF analogue cameras are incorporated in handheld devices, emitting polarized, respectively, stroboscopic green light, with a wavelength within the absorption spectrum of haemoglobin, thereby depicting erythrocytes as black cells. The area of visualization is approximately 1 mm^2^. These techniques are described in detail elsewhere [2, 3]. Offline computer assisted analysis yields information on both convection and diffusion. Microvascular flow index (MFI) is calculated to describe convection in a semiquantitative way; the predominant flow in all quadrants of the SDF/OPS image is scored for different vessel sizes, using a scale ranging from 0 (no flow) to 3 (continuous flow). The averaged flow score yields the MFI for each image. MFI has been shown to correlate well with red blood cell velocity [36]. To obtain information on diffusion, several measures of functional capillary density are calculated, using a grid dividing the image into 16 segments. Every vessel crossing the grid is counted; furthermore, for each vessel crossing the grid, the type of flow using the MFI scale is used, a flow of 0 (no flow) or 1 (intermittent flow) is considered as nonperfused, whereas a flow of 2 (sluggish) or 3 (continuous) describes perfused vessels. By using these data, several measures of functional capillary density can be calculated, including proportion of perfused vessels (PPVs) and perfused vessel density (PVD). Dividing the numbers of perfused grid crossings by the total number of grid crossings yields the PPV; the PVD is calculated as the number of perfused grid crossings divided by the total grid length. In the same way, total vessel density (TVD) can be calculated. A detailed description of MFI and measures of functional capillary density can be found elsewhere [34, 35].

### 2.5. Data Collection

The sublingual microcirculation was measured once in every patient. In line with internationally accepted consensus, 3 to 5 stable sublingual microcirculatory image sequences of 10–20 seconds were obtained for every patient [34, 35]. Along with the SDF/OPS imaging, data on demographics, reason for ICU admission, illness severity scores, haemodynamics, laboratory values, and treatment were collected. Afterwards, information on ICU/hospital length of stay and ICU/hospital mortality will be collected.

### 2.6. Internet-Based Study Equipment

The specifications of the internet platform which has been designed included (1) compliance with international guidelines on clinical research and data security, (2) fast and reliable uploading of clinical data and SDF/OPS images, and (3) facilitating adequate two-way communication. To facilitate communication, an e-mail server and an open access website (http://www.microcirculationstudies.org) have been developed. The website provided general study information and included a weblog and a frequently asked questions section to keep participants updated on the latest study news. For data exchange, a dedicated database has been developed, based on Open Clinica (OC) 3.1 open source (GNU LPGL license) clinical trial software [37]. OC is, amongst others, in compliance with 21 CFR Part 11 (FDA), ICH-GCP and the US Health Insurance Portability and Accountability Act of 1996 (HIPAA). It is a Java J2EE-based application that runs on both Linux and Windows servers. Several other large multicenter studies have used OC databases, for example, the European Surgical Outcomes Study (EuSOS) and the Fluid Expansion As a Supportive Therapy (FEAST) trial [38, 39]. OC allows customization of its database to meet study requirements. The electronic CRF is defined using a special Excel sheet, which is uploaded to the server to define the CRF in the database. For the OC database, a dedicated server is available. Every participating center can log in to a part of the database that is assigned to their ICU to fill out the electronic CRF. After completion of data collection, data will be exported to SPSS 18.0, IBM, New York, USA, for statistical analysis.

A USB stick has been provided to each center with software developed specifically for this study. Its purpose is to provide the user a film editor so that captured film fragments can be edited to identify suitable clips for submission to the study center in Leeuwarden and to provide the needed communication protocols with the servers. The raw SDF/OPS image file can be imported in the image-editing program. By playing the SDF/OPS video, the user will be able to set a start mark and end mark at the appropriate points of the SDF/OPS clip, defining the part of the raw SDF/OPS file that will be used for subsequent analysis. The limit for the maximum clip length is set at 500 frames, that is, 20 seconds. In that way, we will be able to look for the part of the clip that is most suitable for analysis. The software on the USB stick automatically establishes an Internet connection with the central dedicated microSOAP study server using the required communication protocol and security settings. Backup copies of the clips are automatically stored on the USB stick. In case of failure of Internet connection, the USB stick containing the backup clips can alternatively be sent to the study coordinator by regular mail.

### 2.7. Data Analysis

#### 2.7.1. Sample Size Calculation

Because this is the first extensive prevalence study on microcirculatory alterations ever done, with a primarily explorative character, a concise power calculation is virtually impossible. Based on previous studies, with a sample size between 25 and 50 patients, in heart failure, high-risk noncardiac surgery, sepsis, and paediatric ICU patients, significant correlations between the existence of microcirculatory alterations and parameters of morbidity and mortality could be established [6, 10, 12]. However, it is reasonable to assume that morbidity and mortality may be lower in a general ICU population. Therefore, we aim for a sample size ten times larger than previously reported in single-center subgroup studies. Since this is by far the largest cohort of in vivo microcirculatory research in humans ever done, practical limitations with respect to availability of SDF/OPS technique and skilled operators will undoubtedly play a significant role in the definitive sample size.

#### 2.7.2. SDF/OPS Image Analysis

The SDF/OPS image analysis will be performed by the researchers appointed by the initiators of this study in accordance with internationally accepted guidelines using dedicated software [34, 35]. The analysis will be conducted blinded to the origin of the film clips. In a suitable subgroup, an automatic assessment method will be performed as described elsewhere to investigate the suitability of such an automatic software for evaluation and quantification of microcirculatory alterations [40].

Due to the demanding imaging technique, quality of SDF images may vary between centers [41]. However, up-to-date externally validated image quality scoring systems appear to be lacking. To ensure consistency in SDF analysis, SDF analysis will be performed by researchers appointed by the principal investigators, taking care for ongoing feedback and aiming for consensus. Since several reports from different research groups have reported excellent inter- and intraobserver agreement for the SDF image analysis, the steering committee decided beforehand that this would not be an extra topic of this study [5, 10, 34].

#### 2.7.3. Statistical Analysis

Descriptive statistics will be used to describe the study population. Further statistical analysis will be conducted to relate the microcirculatory alterations to the severity of disease and other parameters. The primary outcome measure is the prevalence of microcirculatory alterations. There is no consensus about the thresholds for a “normal” and an “abnormal” microcirculation. Several researchers have reported on values of several microcirculatory variables in healthy volunteers; MFI of capillaries (<20 *μ*m) is reported to be 3.0 [2.9–3.0] (median [IQR]), 2.82 (0.1), and 2.97 (0.03) (mean(SD)) (IQR = interquartile range, SD = standard deviation) [5, 11, 42]. Therefore, one expects 95% of healthy subjects to have a small vessel MFI between 2.62 and 3 [11]. In healthy volunteers, PPVs (small vessels) well above 90% are described, whereas in septic patients, a capillary PPV of 78% (23%) is described [11, 42, 43]. In septic shock, norepinephrine dose >0.1 microgram/kg/min and a lactate >2 mmol/L were associated with a significantly lower PVD (12 [8–15] versus 14 [11–17] n/mm^2^ for norepinephrine dose >0.1 microgram/kg/min and 10 [8–13] versus 14 [11–17] n/mm^2^ for lactate >2 mmol/L), as well as a significantly lower PPV (80 [70–91] versus 100 [90–100]% for norepinephrine dose >0.1 microgram/kg/min and 82 [71–99] versus 93 [84–100]% for lactate >2 mmol/L) [44]. In uncomplicated major abdominal surgery, preoperative PPV (small vessels) was 89% (83–95) versus 79% (73–92) in patients who developed complications postoperatively [4]. In this study, ROC curves will be used to find cut off values of microcirculatory variables in relation to morbidity and mortality.

Secondary outcome parameters are the correlation between microcirculatory changes and macrohaemodynamic variables, correlations between microcirculatory changes, and length of ICU/hospital stay, mortality, and SOFA/APACHE II scores [45, 46]. Differences between several subgroups will be assessed using a *t*-test in case of normally distributed variables; in case of non-normally distributed variables, a nonparametric test will be chosen. Whenever applicable, forward stepwise logistic regression analysis will be used to test for associations between the severity of microcirculatory dysfunction and illness severity, mortality, and length of stay. In addition, the relation between microcirculatory alterations, applied therapy (e.g., fluid therapy and vasopressor therapy), and indicators of peripheral perfusion (e.g., lactate) will be explored. Furthermore, the geographical distribution of microcirculatory alterations will be assessed.

## 3. Discussion

This study will be by far the largest cohort of in vivo microcirculation research. We aim to provide insight in the worldwide prevalence and distribution of microcirculatory alterations. The questions we hope to answer are the following:

1. Does the presence of microcirculatory alterations indicate impending bad outcome in terms of morbidity and mortality?
2. Does the presence of microcirculatory alterations provide a more sensitive indicator of morbidity and mortality then conventional hemodynamic and oxygen derived parameters?
3. Is the presence of microcirculatory alterations related to applied therapy, such as fluid therapy and vasopressors?
4. Is there a difference between microcirculatory alterations in different patient (sub-) groups, and how are these geographically distributed, as well as over time?

We expect that the results of our study will make clinicians more aware of the presence and importance of microcirculatory alterations in daily practice, thereby leading to better identification of patients who are at risk of an unfavorable outcome. Furthermore, we hope to trigger researchers to develop methods enabling easier bedside evaluation of the microcirculation for detection of those at risk of “microcirculatory failure”, as well as interventions aimed at ameliorating the microcirculation. Hopefully, by putting the microcirculation in a central position in future ICU practice, outcome of critically ill patients will be improved.

## 4. Conclusion

With an anticipated inclusion rate of approximately 500 patients, this study will provide the largest reported database of clinical in vivo microscopy in critically ill patients. We expect that this study will form a solid basis for a deeper understanding of the prevalence and meaning of microcirculatory alterations in intensive care patients and show the way forward to the design of a goal-directed interventional study based on the normalization of microcirculatory alterations in intensive care patients.
